# Immune Checkpoint Inhibitor‐Related Myocarditis: A Single Center Observational Registry

**DOI:** 10.1002/clc.70154

**Published:** 2025-05-22

**Authors:** Grace Alexander, Ibrahim Mortada, Mohammed Mhanna, Stefano Byer, Udhayvir Singh Grewal, Shareef Mansour

**Affiliations:** ^1^ Department of Medicine University of Iowa Hospitals & Clinics Iowa City Iowa USA; ^2^ Department of Medicine, Division of Cardiology University of Iowa Hospitals & Clinics Iowa City Iowa USA; ^3^ Department of Medicine, Division of Hematology, Oncology, and Blood & Marrow Transplantation University of Iowa Hospitals & Clinics Iowa City Iowa USA

**Keywords:** cardio‐oncology, immune checkpoint inhibitor, myocarditis

## Abstract

**Introduction:**

Immune checkpoint inhibitors (ICIs) are associated with myocarditis, which is rare but has a high mortality. This study aimed to describe cases of ICI‐related myocarditis at the University of Iowa Hospitals & Clinics and, in doing so, provide valuable insights into patient characteristics, treatment, and outcomes.

**Methods:**

This single‐center observational registry included cases of ICI‐related myocarditis identified from 2009 to 2024. Data were collected retrospectively from electronic medical records and included demographics, cardiovascular risk factors, medications, and cancer characteristics. Between‐group comparisons for continuous data were conducted using unpaired Student's *t*‐tests or the Wilcoxon rank‐sum test. Categorical data were analyzed with Fisher's exact test.

**Results:**

Eighteen patients were included. The mean age was 74 ± 9.4 years with 61% being male. Compared to controls, patients with ICI‐related myocarditis had a significantly higher prevalence of coronary artery disease (36.8% vs. 7.5%, *p* = 0.01) and obstructive sleep apnea (33% vs. 10%, *p* = 0.03). They were less likely to have a normal sinus rhythm on baseline electrocardiogram (50% vs. 70%, *p* < 0.01) and more likely to suffer from a major adverse cardiac event (MACE) (38.9% vs. 2.5%, *p* < 0.01). Twelve (66.7%) of patients with ICI‐related myocarditis also had myasthenia gravis‐like overlap syndrome and 9 (50%) had myositis/rhabdomyolysis.

**Conclusions:**

ICI‐related myocarditis at a tertiary care center is rare with a calculated incidence of 0.48%. Despite this, the disease has a high incidence of MACE. Patients with pre‐existing cardiovascular disease are at higher risk of developing ICI‐related myocarditis. Careful cardiovascular monitoring in patients undergoing ICI therapy is warranted.

## Graphical abstract

In this retrospective case‐control study, patients with coronary artery disease, obstructive sleep apnea, or conduction abnormalities had higher odds of developing immune checkpoint inhibitor (ICI)‐related myocarditis. Those with ICI‐related myocarditis were more likely to experience major adverse cardiac events, in‐hospital mortality, and myasthenia overlap syndromes.

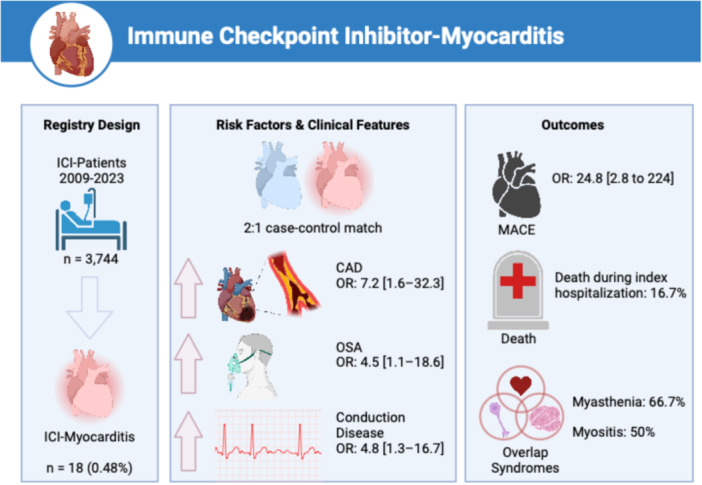

**Abbreviations**
ACE: angiotensin converting enzyme
CAD: coronary artery disease
ECG: electrocardiogram
ICI: immune checkpoint inhibitor
IrAE: immune related adverse event
MACE: major adverse cardiac event
MRI: magnetic resonance imaging
OSA: obstructive sleep apnea

## 1 Introduction

Immune checkpoint inhibitors (ICIs) have revolutionized the field of oncology and are now standard of care for the management of a variety of different cancers. As ICIs become more widely used, immune related adverse events (IrAEs) are increasingly being described in the literature. IrAEs can range in severity from mild to life‐threatening, and can involve virtually any organ system.

While ICI‐related myocarditis is rare, with an estimated incidence of 0.27% [1] to 1.14% [2], it is worth investigating due to the high mortality which is reportedly up to 50% [2, 3, 4, 5] despite treatment with high‐dose steroids or immunomodulating agents which are standard of care. The clinical presentation can range from asymptomatic troponin elevations to cardiogenic shock and life‐threatening arrhythmias. Diagnosis requires a high index of suspicion and multiple diagnostic modalities, including electrocardiogram (ECG), echocardiography, serum biomarkers, and often cardiac magnetic resonance imaging (MRI) and endomyocardial biopsy [6, 7]. The management of ICI‐related myocarditis includes holding the offending immune‐checkpoint inhibitor, high‐dose corticosteroids, guideline‐directed medical therapy for associated heart failure or arrhythmias [6], as well as other steroid‐sparing immunomodulators in steroid‐intolerant or refractory cases. This study aimed to describe cases of ICI‐related myocarditis at the University of Iowa Hospitals & Clinics, comparing them with a cohort of controls, and in doing so, provide valuable insights into patient characteristics, treatment, and outcomes.

## 2 Methods

### 2.1 Study Design and Patient Selection

This study was conducted as a single‐center observational registry comprising cases of ICI‐related myocarditis identified from January 1, 2009 to November 29, 2023. Eligible patients included those with a diagnosis of myocarditis following treatment with ICIs, confirmed by clinical presentation, biomarkers, imaging, and/or histology. The control group consisted of patients receiving ICI therapy who did not develop myocarditis, matched in a [2:1] ratio. This study was approved by the University of Iowa Hospitals & Clinics Institutional Review Board (IRB#202003670). Given the retrospective nature of the chart review and absence of any intervention, the requirement for individual patient consent was waived. All data were deidentified and securely stored in accordance with institutional policies and regulatory standards to ensure patient confidentiality.

### 2.2 Data Collection

Data were collected retrospectively from electronic medical records and included demographics, cardiovascular risk factors, medications, and cancer characteristics. Myocarditis cases were diagnosed based on elevated cardiac biomarkers, electrocardiographic changes, imaging findings consistent with myocarditis, and/or histopathologic evidence. Cancer‐specific data included primary diagnosis, ICI regimen, prior cancer therapies, and incidence of other immune‐related adverse events. Major adverse cardiac events (MACE), defined as cardiovascular death, cardiac arrest, cardiogenic shock, and significant complete heart block, were recorded as the primary outcome.

### 2.3 Definitions and Endpoints

Myocarditis was diagnosed using criteria including histopathology or guideline‐recommended clinical, biomarker, and imaging findings. MACE was defined as a composite of cardiovascular death, cardiac arrest, cardiogenic shock, and complete heart block, with event severity graded per the Common Toxicity Criteria for Adverse Events (version 4.0).

### 2.4 Statistical Analysis

Continuous variables are presented as mean ± SD or median (interquartile range) based on data distribution, and categorical variables are presented as frequencies and percentages. Between‐group comparisons for continuous data were conducted using unpaired Student's *t*‐tests or the Wilcoxon rank‐sum test. Categorical data were analyzed with Fisher's exact test. Multivariate analyses using the Cox proportional hazards regression model was performed to identify predictors of mortality among patients with ICI‐related myocarditis, as well as predictors of ICI‐related myocarditis among patients receiving ICI therapy. Statistical significance was set at a two‐sided *p*‐value of < 0.05. Analyses were performed using IBM SPSS Statistics, version 24.

## 3 Results

A total of 18 patients were identified as meeting inclusion criteria for ICI‐related myocarditis at our institution, and 40 patients were included in the control group.

### 3.1 Baseline (Pre‐ICI) Characteristics

The mean age of patients with ICI‐related myocarditis (*n* = 18) was 74 ± 9.4 years with 61% being male (Table 1). A majority were Caucasians (95%) with hypertension (78%), a history of smoking (61%), metastatic cancer (72.2%), and no prior history of chemotherapy (83.3%). None of the cases or controls had prior ICI exposure before the most recent therapy. When comparing to controls, patients who developed myocarditis had a significantly higher prevalence of coronary artery disease (CAD) (36.8% vs. 7.5%, OR: 7.2, CI 1.6−32.3, *p* = 0.01) and obstructive sleep apnea (OSA) (33% vs. 10%, OR: 4.5, CI 1.1−18.6, *p* = 0.03). Patients with myocarditis were less likely to have a prior history of chemotherapy exposure (16.7% vs. 50%, OR: 0.2, CI 0.05−0.8, *p* = 0.02). Prior angiotensin converting enzyme (ACE) inhibitor and aspirin use were more common among patients who developed myocarditis (50% vs. 17.5%, OR: 4.7, CI 1.3−16.9, *p* = 0.01; 55.5% vs. 15%, OR: 9.4, CI 2.21−29.67, *p* < 0.01). Patients with myocarditis were significantly less likely to have normal sinus rhythm on baseline ECG (50% vs. 70%, OR 0.21, CI 0.06−0.77, *p* < 0.01). Accelerated junctional rhythm and first‐degree heart block was more commonly noted among patients with myocarditis. Average baseline left ventricular ejection fraction for cases and controls was grossly normal at 60 ± 5.9 and 57 ± 7.5, respectively (*p* = 0.45).

**Table 1:** Description of cases and controls.

	Myocarditis (*n* = 18)	Controls (*n* = 40)	Odds ratio	95% CI	*p* value
Age at start of ICI (years)	74 ± 9.4	69.7 ± 9.2	4.5	−0.78, 9.7	0.09
Male	11 (61)	21 (52.5)	0.70	0.23, 2.2	0.54
Caucasian	17 (95)	39 (97.5)	0.44	0.03, 7.4	0.55
BMI (kg/m^2^)	28.5 ± 5.9	29.3 ± 9	−0.73	−4.8, 3.3	0.75
CV risk factors					
Current or prior smoking	11 (61)	27 (67.5)	0.76	0.24, 2.4	0.64
Hypertension	14 (78)	26 (65)	1.9	0.52, 6.8	0.33
Diabetes mellitus	5 (28)	8 (20)	1.5	0.4, 5.4	0.54
Coronary artery disease^a^	6 (33.3)	3 (7.5)	6.2	1.3, 28.5	0.01
Heart failure	0 (0)	1 (2.5)			0.49
Stroke	1 (5.6)	2 (5)	1.1	0.09, 13.2	0.93
Chronic obstructive pulmonary disease	2 (11)	7 (17.5)	0.59	0.11, 3.16	0.53
Obstructive sleep apnea	6 (33)	4 (10)	4.5	1.1, 18.6	0.03
Chronic kidney disease^b^	4 (22)	6 (15)	1.6	0.39, 6.63	0.50
Primary cancer type					
Urothelial carcinoma	3 (15)	4 (10)	1.2	0.26, 5.78	0.63
Cervical cancer	1 (5.6)	1 (2.5)	2.3	0.13, 38.9	0.55
Cholangiocarcinoma	0 (0.0)	1 (2.5)			0.49
Colorectal cancer	0 (0.0)	1 (2.5)			0.49
Endometrial cancer	0 (0.0)	4 (10)			0.16
Esophageal cancer	1 (5.6)	2 (5.0)	1.1	0.09, 12.8	0.95
Hepatocellular carcinoma	0 (0.0)	1 (2.5)			0.49
Hodgkin's lymphoma	0 (0.0)	1 (2.5)			0.49
Renal cell carcinoma	0 (0.0)	7 (17.5)			0.06
Leukemia	0 (0)	1 (2.5)			0.49
Lung cancer	3 (16.7)	10 (25)	0.60	0.14, 2.51	0.51
Melanoma	6 (33.3)	5 (12.5)	3.5	0.90, 13.6	0.06
Thymoma	1 (5.6)	0 (0.0)			0.13
Other cancer	4 (22.2)	2 (5.0)	5.4	0.89, 32.9	0.05
Prior chemotherapy or radiation					
Radiation	6 (33.3)	11 (27.5)	1.32	0.39, 4.38	0.65
Chemotherapy	3 (16.7)	20 (50)	0.20	0.05, 0.80	0.02
Pre‐ICI echocardiogram					
LVEF, %	60 ± 5.9	57 ± 7.5	2.7	−4.8, 10.3	0.45
Left ventricular internal dimension in diastole, mm	46 ± 5.6	44 ± 5.4	1.95	−3.9, 7.9	0.50
Pre‐ICI electrocardiogram					
Sinus rhythm	9 (50)	28 (70)	0.21	0.06, 0.77	< 0.01
Accelerated junctional	1 (5.6)	0 (0.0)			0.16
First‐degree heart block	2 (11.1)	0 (0.0)			0.047
Heart rate, beats/min	73 ± 17	75 ± 14	−2.3	−12.3, 7.8	0.65
PR interval, ms	188 ± 74	156 ± 23	32.4	3.2, 61.5	0.03
QRS interval, ms	97.5 ± 27.8	97.5 ± 24.2	0	−17, 17	1.00
QTc interval, ms	433 ± 25	440 ± 21	−6.8	−22, 8.4	0.37
Pre‐ICI home CV medications					
Beta‐blockers	3 (16.6)	10 (25)	0.69	0.16, 2.94	0.62
Angiotensin‐converting enzyme inhibitor or angiotensin receptor blocker	9 (50)	7 (17.5)	4.7	1.3, 16.9	0.01
Mineralocorticoid receptor antagonists	0 (0.0)	6 (15)			0.10
Sodium‐glucose cotransporter 2 inhibitors	0 (0.0)	1 (2.5)			0.54
Loop diuretics	0 (0.0)	3 (7.5)	0.82	0.08, 8.54	0.27
Thiazide diuretics	3 (16.6)	5 (12.5)	1.57	0.33, 7.52	0.57
Aspirin	10 (55.5)	6 (15)	9.4	2.5, 35.8	< 0.01
Statin	10 (56.3)	15 (37.5)	2.1	0.66, 6.95	0.20

*Note:* Values are mean ± SD or *n* (%), unless otherwise indicated.

Abbreviations: CV = cardiovascular, LVEF = left ventricular ejection fraction.

a Coronary artery disease is defined as a previous acute myocardial infarct, angina, percutaneous coronary intervention, or coronary artery bypass graft.

b Chronic kidney disease is defined as a glomerular filtration rate of < 60 mL/min per 1.73 m.^2^

### 3.2 Cancer Treatment

Anti‐PD‐1 monotherapy comprised the most common type of ICI therapy among both cases and controls (88.9% and 87.5%, Supporting Information S1: Table S4). Pembrolizumab specifically was the most common agent among cases (65%) and controls (50%). Nivolumab was the second most common agent among cases (22.2%), with significant differences in mean dose per cycle between the two groups (330 ± 114.9 vs. 40.5 ± 117 mg, *p* < 0.01). Patients had a variety of different cancers. Among cases of myocarditis, melanoma was the most common indication for ICI therapy (30%), whereas lung cancer was the most common among controls (25%). Only one patient among the cases received combination ICI therapy with nivolumab and relatlimab.

### 3.3 Presentation of IrAE

ICI‐related myocarditis was classified as severe G4 (*n* = 6), moderate G3 (*n* = 6), mild G2 (*n* = 3), and asymptomatic G1 (*n* = 3). Twelve (66.7%) of patients with ICI‐related myocarditis also had myasthenia gravis‐like overlap syndrome and 9 (50%) had myositis/rhabdomyolysis (Supporting Information S1: Table S4). A variety of serious complications were significantly more common among myocarditis cases compared to controls. Respiratory failure, acute respiratory distress syndrome, diaphragmatic failure, immunotherapy‐induced pneumonitis, and pulmonary infection were each found in 6 (33.3%) of patients with myocarditis. MACE occurred in seven of the 18 myocarditis cases, which is significantly higher than in cases (38.9% vs. 2.5%, *p* < 0.01). Of the myocarditis cases with MACE, two had heart failure, one had arterial thrombosis, four had life‐threatening arrhythmia, and one had pulmonary embolism.

### 3.4 Outcome and Incidence

Three (16.7%) of the 18 myocarditis cases died within the current hospital stay and 10 (55.6%) had died after the hospitalization at the time of review. The most common cause of death among ICI‐related myocarditis cases was cancer progression (55.6%), followed by IrAEs other than cardiotoxicity (27.8%) and respiratory failure (27.8%). Death attributed to myocarditis was less common (16.7%). Regarding incidence, the total number of patients who received therapy with ICIs from 2009 to 2023 at our institution was 3744, therefore our calculated incidence of ICI myocarditis is 0.48%.

### 3.5 Comparison of Myocarditis Cases With and Without MACE

There were significantly more males in the MACE group (7 vs. 4, *p *< 0.01), otherwise baseline characteristics did not significantly differ (Supporting Information S1: Table S5). Mean left ventricular ejection fraction on presentation was lower in cases with MACE compared to those without MACE, 46.8 ± 20 versus 58.2 ± 7.6, though not reaching significance (*p *= 0.12); however left ventricular end‐diastolic diameter differed significantly between the two groups (48.4 ± 5.8 vs. 43.7 ± 3.6, *p* = 0.04). Mean serum creatinine, both initial and peak, were significantly higher in cases with MACE compared to those without MACE (*p *< 0.01). Additionally, the N‐terminal prohormone brain natriuretic peptide (NT‐proBNP) followed a similar trend between groups for both initial and peak values (Supporting Information S1: Table S5). There were no significant difference in vital signs on presentation (Supporting Information S1: Table S5). Congestive heart failure and dysrhythmias were both significantly more common among cases with MACE compared to those without. There were no significant differences in noncardiac IrAEs such as myasthenia gravis‐like syndrome and myositis/rhabdomyolysis (Supporting Information S1: Table S5). Regarding clinical presentations, prolonged dyspnea before presentation (4.6 ± 2.0 vs. 1 ± 1.0 days) was more common among cases with MACE (*p* < 0.01). There were no significant differences between groups for treatment with the exception of temporary pacemaker placement being more common in the MACE group (*p* = 0.02).

### 3.6 Predictors of Mortality and ICI‐Related Myocarditis

Table 2 presents the results of a multivariable analysis evaluating clinical and biomarker variables as potential predictors of mortality in patients diagnosed with ICI‐related myocarditis. None of the variables demonstrated a statistically significant association with mortality. Similarly, Table 3 displays the results of a multivariable analysis evaluating potential predictors of developing ICI‐related myocarditis among patients receiving ICI therapy. The variables assessed include demographic factors, cardiovascular comorbidities, medication use, ICI regimen type, and baseline cardiac function. None of the evaluated variables reached statistical significance.

**Table 2:** Predictors of mortality among patients with ICI myocarditis.

Variable	HR	95% CI	*p* value
Age	0.43	0.1−1.8	0.25
Gender (male)	1.3	0.1−1.4	0.21
CAD	1.1	0.01−1.2	0.51
LVEF	1.3	0.03−49.9	0.91
CK level	0.99	0.98−1.0	0.14
NT‐pro‐BNP	1.01	0.99−1.02	0.55
HsTrp	1.01	0.95−1.09	0.60

Abbreviations: CAD = coronary artery disease, CK = creatinine kinase, HsTrp = high sensitivity troponin, LVEF = left ventricular ejection fraction, NT‐pro‐BNP = N‐terminal pro‐B‐type natriuretic peptide.

**Table 3:** Predictors of ICI‐related myocarditis among patients treated with ICI.

Variable	HR	95% CI	*p* value
Age	0.98	0.84−1.14	0.81
Gender (male)	2.5	0.01−61.6	0.52
CAD	3.6	0.01−25.0	0.53
ASA use	0.01	0.001−14.6	0.14
BB use	7.1	0.03−17.8	0.48
Anti‐PD‐1	5.5	0.001−53.8	0.88
Anti‐PD‐L1	1.1	0.001−1.99	0.88
ICI combination	0.4	0.03−4.9	0.48
Baseline LVEF	0.94	0.85−1.03	0.19

Abbreviations: ASA = aspirin, BB = beta‐blocker, CAD = coronary artery disease, ICI = immune checkpoint inhibitor, LVEF = left ventricular ejection fraction.

## 4 Discussion

This study provides a detailed analysis of 18 cases of ICI‐related myocarditis diagnosed at an institution over a 14.8‐year period. Our findings reveal that patients with ICI‐related myocarditis exhibit distinct baseline characteristics, clinical presentations, and outcomes compared to a control group of 40 patients receiving ICI therapy. Based on univariate analysis, patients with myocarditis were significantly more likely to have CAD and OSA, and they were less likely to have a history of prior chemotherapy. They also had higher rates of baseline ECG abnormalities and were more frequently on ACE inhibitors and aspirin. Although prior research has shown an association between ICI‐related myocarditis and concomitant diuretic use [8], our study found no difference in rates of loop or thiazide diuretics among cases and controls. The higher prevalence of CAD and OSA in patients with ICI‐related myocarditis suggests that underlying cardiovascular conditions may predispose patients to myocarditis when undergoing ICI therapy. The association with ACE inhibitors and aspirin raises the question of whether these medications might influence the development of myocarditis or if they are merely reflective of pre‐existing cardiovascular conditions. In the multivariable analyses, we did not identify significant clinical or biomarker predictors of either the development of ICI‐related myocarditis or mortality among affected patients. While variables such as CAD, β‐blocker use, and ICI regimen type showed a trend toward association with myocarditis, none reached statistical significance. These findings likely reflect the limited statistical power and highlight the need for larger studies to identify reliable risk and prognostic factors in ICI‐related myocarditis.

ICI myocarditis is characterized by inflammation of the myocardium, thus leading to electrophysiological abnormalities such as second and third‐degree atrioventricular blocks [9, 10]. Our study found that patients who develop myocarditis have a significantly higher prevalence of 1st degree heart block on baseline ECG compared to controls. This finding suggests that pre‐existing conduction disease is a risk factor for developing cardiotoxicity from ICIs. Furthermore, it highlights the need for vigilant cardiovascular monitoring in patients with underlying ECG abnormalities receiving ICI therapy. Along the same lines of monitoring and prevention, our results suggest that lab abnormalities such as elevated creatinine and NT‐proBNP could be used to risk‐stratify ICI‐related myocarditis cases on presentation, identifying those that may benefit from closer monitoring and prompt intervention.

Our study found significant overlap between myocarditis cases and myasthenia‐gravis‐like syndrome (66.7%) and myositis/rhabdomyolysis (50%). This is higher than other estimations that as many as 25% of myocarditis cases can involve skeletal muscle [11]. Clinicians should have a high index of suspicion for other muscle involvement when treating ICI myocarditis. The combination of myocarditis, myositis, and myasthenia gravis occurring as an overlap syndrome is important to be aware of because of its significant mortality, reported as high as 60% in a collection of 60 cases [12].

There are limitations which should be acknowledged to fully understand the findings of this study. First, this was a single‐center, retrospective case‐control study at a tertiary care center which limits the generalizability of results and also makes the population of cases more susceptible to referral bias, attracting increased complexity of cases. Second, selection bias is inherent in a case series methodology. Finally, our sample size is small, and we may have missed cases that SlicerDicer did not capture. Of note, despite these limitations, this study does offer a detailed analysis of the clinical features of patients, and this is one advantage compared to ICI‐related myocarditis studies that rely on adverse event databases.

## 5 Conclusion

This study offers important insights into the characteristics, clinical presentation, and outcomes of patients with ICI‐related myocarditis. ICI‐related myocarditis is rare but associated with a high incidence of MACE. Our findings support the need for careful cardiovascular monitoring in patients undergoing ICI therapy, particularly those with pre‐existing conduction disease, CAD, or OSA. Future research should aim to explore these findings in larger, multicenter studies and investigate the underlying mechanisms linking pre‐existing conditions with the development of ICI‐related myocarditis. Prospective studies with standardized protocols for cardiac monitoring and management may help refine guidelines and improve outcomes for patients undergoing ICI therapy. The insights gained from this research can guide future studies and improve patient management, ultimately enhancing the safety and efficacy of ICI therapies.

## Disclosure

The authors have nothing to report.

## Conflicts of Interest

The authors declare no conflicts of interest.

## Supporting information

Supplemental.

## Data Availability

The data that support the findings of this study are available from the corresponding author upon reasonable request.

## References

[1] D. B. Johnson , J. M. Balko , M. L. Compton , et al., “Fulminant Myocarditis With Combination Immune Checkpoint Blockade,” New England Journal of Medicine 375, no. 18 (2016): 1749–1755. 27806233 10.1056/NEJMoa1609214 PMC5247797

[2] S. S. Mahmood , M. G. Fradley , J. V. Cohen , et al., “Myocarditis in Patients Treated With Immune Checkpoint Inhibitors,” Journal of the American College of Cardiology 71, no. 16 (2018): 1755–1764. 29567210 10.1016/j.jacc.2018.02.037 PMC6196725

[3] D. Y. Wang , J.‐E. Salem , J. V. Cohen , et al., “Fatal Toxic Effects Associated With Immune Checkpoint Inhibitors: A Systematic Review and Meta‐Analysis,” JAMA Oncology 4, no. 12 (2018): 1721. 30242316 10.1001/jamaoncol.2018.3923 PMC6440712

[4] J.‐E. Salem , A. Manouchehri , M. Moey , et al., “Cardiovascular Toxicities Associated With Immune Checkpoint Inhibitors: An Observational, Retrospective, Pharmacovigilance Study,” Lancet Oncology 19, no. 12 (2018): 1579–1589. 30442497 10.1016/S1470-2045(18)30608-9 PMC6287923

[5] J. J. Moslehi , J.‐E. Salem , J. A. Sosman , B. Lebrun‐Vignes , and D. B. Johnson , “Increased Reporting of Fatal Immune Checkpoint Inhibitor‐Associated Myocarditis,” Lancet 391, no. 10124 (2018): 933. 10.1016/S0140-6736(18)30533-6 PMC6668330 29536852

[6] N. Palaskas , J. Lopez‐Mattei , J. B. Durand , C. Iliescu , and A. Deswal , “Immune Checkpoint Inhibitor Myocarditis: Pathophysiological Characteristics, Diagnosis, and Treatment,” Journal of the American Heart Association 9, no. 2 (2020): e013757. 31960755 10.1161/JAHA.119.013757 PMC7033840

[7] J. R. Brahmer , C. Lacchetti , B. J. Schneider , et al., “Management of Immune‐Related Adverse Events in Patients Treated With Immune Checkpoint Inhibitor Therapy: American Society of Clinical Oncology Clinical Practice Guideline,” Journal of Clinical Oncology 36, no. 17 (2018): 1714–1768. 29442540 10.1200/JCO.2017.77.6385 PMC6481621

[8] S. Mitsuboshi , H. Hamano , T. Niimura , et al., “Association Between Immune Checkpoint Inhibitor‐Induced Myocarditis and Concomitant Use of Thiazide Diuretics,” International Journal of Cancer 153, no. 8 (2023): 1472–1476. 37306521 10.1002/ijc.34616

[9] W. Song , Y. Zheng , M. Dong , et al., “Electrocardiographic Features of Immune Checkpoint Inhibitor‐Associated Myocarditis,” Current Problems in Cardiology 48, no. 2 (2023): 101478. 36336121 10.1016/j.cpcardiol.2022.101478

[10] J. R. Power , J. Alexandre , A. Choudhary , et al., “Electrocardiographic Manifestations of Immune Checkpoint Inhibitor Myocarditis,” Circulation 144, no. 18 (2021): 1521–1523. 34723640 10.1161/CIRCULATIONAHA.121.055816 PMC8567307

[11] Y.‐T. Huang , Y.‐P. Chen , W.‐C. Lin , W.‐C. Su , and Y.‐T. Sun , “Immune Checkpoint Inhibitor‐Induced Myasthenia Gravis,” Frontiers in Neurology 11 (2020): 634. 32765397 10.3389/fneur.2020.00634 PMC7378376

[12] R. Pathak , A. Katel , E. Massarelli , V. M. Villaflor , V. Sun , and R. Salgia , “Immune Checkpoint Inhibitor–Induced Myocarditis With Myositis/Myasthenia Gravis Overlap Syndrome: A Systematic Review of Cases,” Oncologist 26, no. 12 (2021): 1052–1061. 34378270 10.1002/onco.13931 PMC8649039

