# Supplementary material for: Immune Checkpoint Inhibitor‐Related Myocarditis: A Single Center Observational Registry
Source: Clin Cardiol. 2025 May 22;48(5):e70154. doi: 10.1002/clc.70154 (PMC12096086; doi:10.1002/clc.70154)
Supplement: Supplementary file 1 — Supplemental. [file CLC-48-e70154-s001.docx]

**Table S4. Cancer treatment and clinical course**

| Variable | Myocarditis (n=18) | | Control (n=40) | OR or Mean difference | 95%CI | P-value |
| --- | --- | --- | --- | --- | --- | --- |
| **Anti-cancer therapy the patient was receiving at time of enrollment** | | | | | | |
| Chemotherapy | 4 (22.2) | | 47.5% | 0.32 | 0.09, 0.1.12 | 0.07 |
| Immune checkpoint inhibitor |  | |  |  |  |  |
| Anti-PD-1 | 16 (88.9) | | 35 (87.5) | 1.1 | 0.20, 6.53 | 0.88 |
| Anti-PD-L1 | 1 (5.6) | | 5 (12.5) | 0.41 | 0.04, 3.8 | 0.42 |
| Anti-CTLA- 4 | 0 (0.0) | | 1 (2.5) |  |  | 0.50 |
| Immunotherapy other than ICI | 22.2% | | 12.5% | 2.0 | 0.47, 8.56 | 0.34 |
| Anti-PD-1 |  | |  |  |  |  |
| Nivolumab | 22.2% | | 12.5% | 2.0 | 0.47, 8.56 | 0.34 |
| Nivo dose | 330±114.9 | | 40.5±117 | 289 | 164, 414 | **<0.01** |
| Pembrolizumab | 65% | | 50% | 2.6 | 0.78, 8.67 | 0.11 |
| Pemb dose | 200 | | 169±141 | 30.8 | -52, 113 | 0.46 |
| Cemiplimab | 0% | | 0% |  |  |  |
| Other PD-1 Inhibitor | 0% | | 5% |  |  | 0.93 |
| Anti-PD-L1 |  | |  |  |  |  |
| Atezolizumab | 0 | | 5% |  |  | 0.33 |
| Avelumab \| | 5.6% | | 0% |  |  | 0.13 |
| Durvalumab | 0% | | 10% |  |  | 0.16 |
| Other PD-L1 Inhibitor | 0% | | 0% |  |  |  |
| Anti-CTLA- 4 |  | |  |  |  |  |
| Ipilimumab | 0 | | 5% |  |  | 0.33 |
| Tremelimumab | 0 | | 0% |  |  |  |
| Other CTLA-4 Inhibitor | 0 | | 0% |  |  |  |
| Combined ICI | 5% | | 0% |  |  | 0.13 |
| Current Chemotherapy agents used | | | | | | |
| Alkylating agents | 22.2% | | 37.5% | 0.47 | 0.13, 1.72 | 0.25 |
| Antimetabolites | 22.2% | | 10% | 2.5 | 0.56, 11.7 | 0.21 |
| Platinum | 0% | | 37.5% |  |  | **<0.01** |
| Topo | 0% | | 7.5% |  |  | 0.23 |
| Her2 | 0% | | 0% |  |  |  |
| VEGF | 5.6% | | 17.5% | 0.28 | 0.03, 2.44 | 0.22 |
| Non-cardiac immune-related adverse events | | | | | | |
| None | 22.2% | 25% | | 0.75 | 0.20, 2.78 | 0.82 |
| Arthritis | 0% | 2.5% | |  |  | 0.50 |
| Guillian-Barre Syndrome | 0% | 2.5% | |  |  | 0.50 |
| Hepatitis | 55.6% | 5% | | 23 | 4.3, 129 | **<0.01** |
| Lymphopenia, Immune-Related | 0% | 2.5% | |  |  | 0.50 |
| Meningitis / Encephalitis / Cerebritis | 5.6% | 0% | |  |  | 0.13 |
| Myasthenia gravis-like syndrome | 66.7% | 0% | |  |  | **<0.01** |
| Myositis / Rhabdomyolysis | 50% | 0% | |  |  | **<0.01** |
| Rash / Dermatologic | 0% | 12.5% | |  |  | 0.12 |
| Venous Thromboembolism | 5.6% | 0% | |  |  | 0.13 |
| Patient admitted to the hospital for one of these adverse events prior to cardiotoxicity hospital admission | 5.6% | 5% | | 1.1 | 0.09, 13.2 | 0.93 |
| MACE, complications | | | | | | |
| Sepsis | 11.1% | 2.5% | | 4.9 | 0.41, 57.6 | 0.17 |
| Respiratory failure | 33.3% | 2.5% | | 19.5 | 2.1, 178 | **<0.01** |
| Acute Respiratory Distress Syndrome | 33.3% | 0% | |  |  | **<0.01** |
| Diaphragmatic Failure | 33.3% | 2.5% | | 19.5 | 2.1, 178 | **0.01** |
| Immunotherapy Induced Pneumonitis | 33.3% | 0% | |  |  | **<0.01** |
| Pulmonary Infection | 33.3% | 0% | |  |  | **<0.01** |
| Other Cause | 27.8% | 2.5% | | 15 | 1.6, 140 | **0.03** |
| MACE | 38.9% | 2.5% | | 24.8 | 2.7, 223.9 | **<0.01** |
| HF | 11.1% | 0% | |  |  | **0.03** |
| Arterial Thrombosis (myocardial infarction, stroke, lower limb ischemia) | 5.6% | 0% | |  |  | 0.13 |
| Life-Threatening Arrhythmias | 22.2% | 2.5% | | 11.1 | 1.1, 108 | **0.01** |
| Pulmonary Embolism | 5.6% | 0% | |  |  | 0.13 |
| In-hospital mortality | 16.7% | 2.5% | | 7.8 | 0.75, 81 | **0.049** |
| Care withdrawal | 12.2% | 2.5% | | 11.1 | 1.1, 108 | **0.01** |
| Discharged to Hospice | 13.3% | 5.3% | | 2.8 | 0.35, 21.7 | 0.32 |
| Did this patient die after this hospitalization? | 55.6% | 10% | | 11.2 | 2.8, 45.2 | **<0.01** |
| Cause of Death | | | | | | |
| Myocarditis | 16.7% | 0% | |  |  | **<0.01** |
| Cancer Progression | 55.6% | 12.5% | | 8.7 | 2.3, 32.7 | **<0.01** |
| Immune Related Adverse Event other than Cardiotoxicity | 27.8% | 0% | |  |  | **<0.01** |
| Other Cause | 11.1% | 0% | |  |  | **0.03** |
| Respiratory Failure | 27.8% | 2.5% | | 15 | 1.6, 140 | **<0.01** |

Data are presented as mean ± SD or %, with ORs and 95% CIs for categorical variables. Significant differences (p<0.05) are bolded.

**Table S5: Comparison of Myocarditis Cases with and without MACE**

| Variable | Myocarditis with MACE =7 | Myocarditis without MACE=11 | P-value |
| --- | --- | --- | --- |
| Baseline characteristics | | | |
| Age | 75.1±7.4 | 73.5±10.7 | 0.74 |
| Male | 7 | 4 | **<0.01** |
| BMI | 26.2±4.8 | 30.0±6.3 | 0.19 |
| Caucasian | 7 | 10 | 0.41 |
| Smoking history | 5 | 6 | 0.47 |
| HLD | 4 | 5 | 0.63 |
| DM | 1 | 4 | 0.31 |
| HTN | 5 | 9 | 0.60 |
| CAD | 2 | 4 | 0.73 |
| HF | 0 | 0 |  |
| COPD | 1 | 1 | 0.73 |
| CKD | 2 | 2 | 0.61 |
| Medications | | | |
| ACEi | 2 | 6 | 0.59 |
| Aldosterone antagonist | 0 | 0 |  |
| BB | 1 | 2 | 0.93 |
| SGLT2i | 0 | 0 |  |
| ASA | 3 | 7 | 0.89 |
| Prior Chemotherapy | 0 | 3 | 0.13 |
| Baseline EKG | | | |
| HR | 73±3.4 | 73.6±19.6 | 0.96 |
| PR interval | 167.3±14.4 | 196.6±86.9 | 0.59 |
| QRSd | 99±10.8 | 97±32.1 | 0.92 |
| QTc | 432.7±17.2 | 433.4±28 | 0.97 |
| ECHO on presentation | | | |
| LVEF | 46.8±20 | 58.2±7.6 | 0.12 |
| LVEDd | 48.4±5.8 | 43.7±3.6 | **0.04** |
| Follow-up ECHO | | | |
| LVEF | 59.3±10.4 | 60.1±8.2 | 0.91 |
| LVEDd | 47.4±4.3 | 44.0±7.4 | 0.58 |
| Laboratory work-up | | | |
| ALT, initial | 246±164 | 150.8±120 | 0.20 |
| ALT, peak | 265.5±147.8 | 186.8±132.6 | 0.29 |
| AST, initial | 325.6±234.5 | 205.7±200.8 | 0.29 |
| AST, peak | 333.5±225.1 | 251.2±221.7 | 0.49 |
| CRP, initial | 3.6±3.4 | 1.8±2.5 | 0.35 |
| CRP, peak | 3.6±3.4 | 2.1±2.5 | 0.41 |
| Creatinine, initial | 1.2±0.4 | 0.8±0.14 | **<0.01** |
| Creatinine, peak | 1.5±0.4 | 0.9±0.2 | **<0.01** |
| CK, initial | 5209±6158.8 | 2052.6±1730.9 | 0.19 |
| CK, peak | 5411±6552.2 | 2053.3±1729.9 | 0.18 |
| NT-proBNP, initial | 3382.8±2954.3 | 802.2±696.2 | **0.03** |
| NT-proBNP, peak | 3484±2889 | 802.7±695.7 | **0.02** |
| Trop, initial | 3590.8±7269.2 | 1089±2205.3 | 0.29 |
| Trop, peak | 4099.8±7124.1 | 1462.3±2787 | 0.28 |
| Vital signs on presentation | | | |
| Temp | 36.4±0.3 | 36.6±0.4 | 0.34 |
| HR | 76.4±11.9 | 80.2±18.4 | 0.63 |
| SBP | 128.7±27.3 | 136.0±15.6 | 0.47 |
| DBP | 71.2±15.5 | 77.6±13.4 | 0.37 |
| RR | 22±7.9 | 18.8±3.6 | 0.26 |
| SpO2 | 95.7±2.2 | 96.4±1.8 | 0.45 |
| Cardiotoxicity type | | | |
| Congestive Heart Failure | 3 | 0 | **0.02** |
| Dysrhythmia | 6 | 0 | **<0.01** |
| NSTEMI | 3 | 3 | 0.49 |
| Pericarditis | 0 | 1 | 0.41 |
| Other iRAE | | | |
| None | 1 | 3 | 0.52 |
| Hepatitis | 4 | 6 | 0.91 |
| Encephalitis / Cerebritis | 0 | 1 | 0.41 |
| Myasthenia gravis-like syndrome | 5 | 7 | 0.73 |
| Myositis / Rhabdomyolysis | 4 | 5 | 0.63 |
| Venous Thromboembolism | 1 | 0 | 0.20 |
| Days to presentation | | | |
| From onset of ocular symptoms | 4±1.6 | 3±2.5 | 0.5 |
| From onset of dyspnea | 4.6±2.0 | 1±1.0 | **<0.01** |
| From onset of myalgia | 4.6±2.0 | 4±4.2 | 0.82 |
| From onset of fatigue | 6.6±2.3 | 9.3±16.1 | 0.71 |
| From onset of dysphagia | 3±1.4 | 2.3±2.0 | 0.72 |
| From onset of MG-like symptoms | 3.8±2.2 | 3.4±2.1 | 0.81 |
| Immune checkpoint inhibitor used | | | |
| Anti-PD-1 | 6 | 10 | 0.73 |
| Anti-PD-L1 | 1 | 0 | 0.20 |
| Anti-CTLA- 4 | 0 | 0 |  |
| Nivolumab | 1 | 3 | 0.52 |
| Nivolumab, dose | 240 | 360±120 | 0.47 |
| Nivolumab, number of doses | 4 | 1.67±0.58 | 0.07 |
| Pembrolizumab | 5 | 8 | 0.84 |
| Pembrolizumab, dose | 200 | 200 |  |
| Pembrolizumab, number of doses | 2±1.2 | 3.25±4.1 | 0.52 |
| Atezolizumab | 0 | 3 |  |
| Avelumab \| | 1 | 0 | 0.20 |
| ICI combination | 0 | 1 | 0.41 |
| Current Chemotherapy agents used | | | |
| Alkylating agents | 3 | 1 | 0.09 |
| Antimetabolites | 3 | 1 | 0.09 |
| VEGF | 0 | 1 | 0.41 |
| Target of these current anti-cancer therapies | | | |
| Bladder Cancer | 2 | 1 | 0.28 |
| Cervical Cancer | 0 | 1 | 0.41 |
| Esophageal Cancer | 1 | 0 | 0.20 |
| Lung Cancer | 2 | 1 | 0.28 |
| Melanoma | 1 | 5 | 0.17 |
| Thymoma | 1 | 0 | 0.20 |
| Other Cancer | 0 | 4 | 0.07 |
| Metastatic | 5 | 8 | 0.95 |
| Treatment | | | |
| MCS used | 0 | 0 |  |
| Vasopressors | 1 | 1 | 0.73 |
| Steroids | 7 | 9 | 0.23 |
| Steroid dose | 490±477.3 | 429±450 | 0.80 |
| Steroid subtype |  |  | 0.58 |
| Methylprednisolone | 6 | 6 |  |
| Prednisone | 1 | 2 |  |
| Dexamethasone | 0 | 1 |  |
| Days from presentation to first steroid dose | 0.83±1.8 | 1.2±1.6 | 0.68 |
| Immunomodulators or Plasmapheresis | 4 | 8 | 0.59 |
| Abatacept | 4 | 2 | 0.31 |
| Days from presentation to initiation of Abatacept | 6.5±2.9 | 14.5±7.8 | 0.12 |
| Ruxolitinib | 3 | 2 | 0.25 |
| IVIG | 4 | 7 | 0.26 |
| Days from presentation to initiation of IVIG | 3.3±2.4 | 5.6±4.8 | 0.40 |
| Temporary pacemaker | 3 | 0 | **0.02** |
| PPM | 2 | 0 | 0.06 |
| ICD | 1 | 0 | 0.20 |
| Antiarrhythmic drugs | 4 | 3 | 0.21 |
